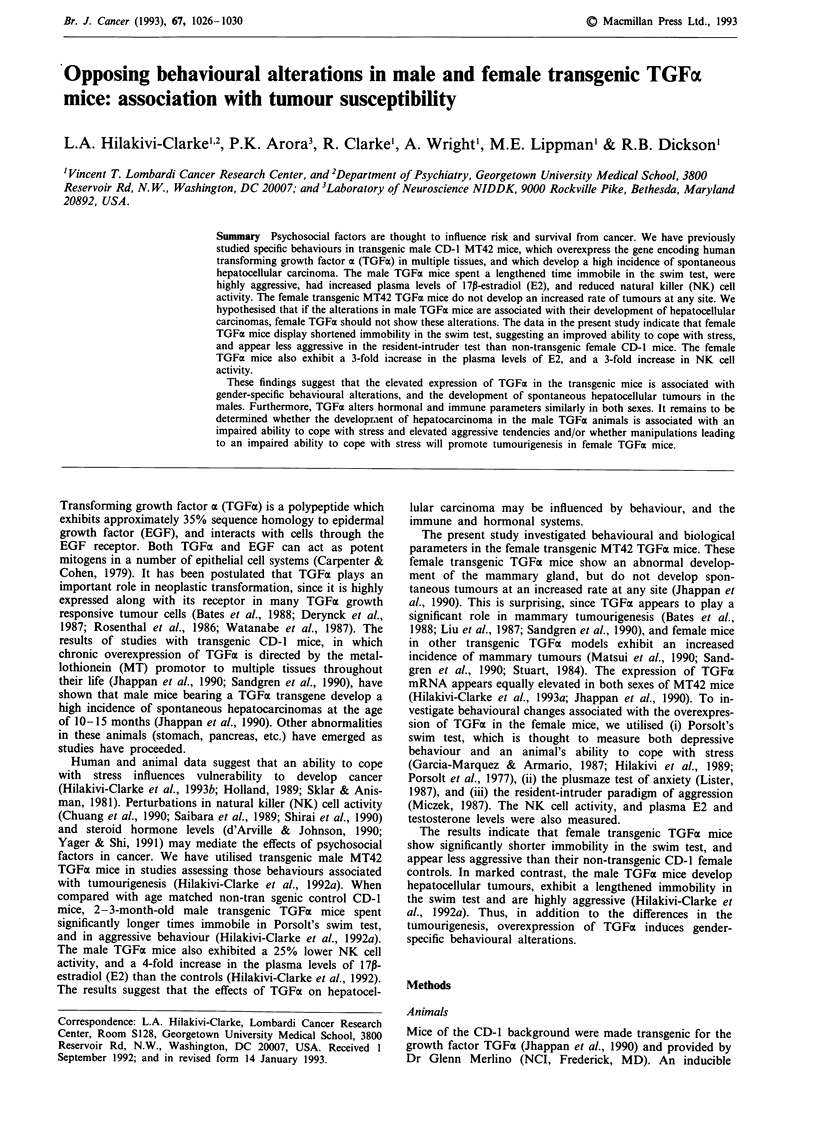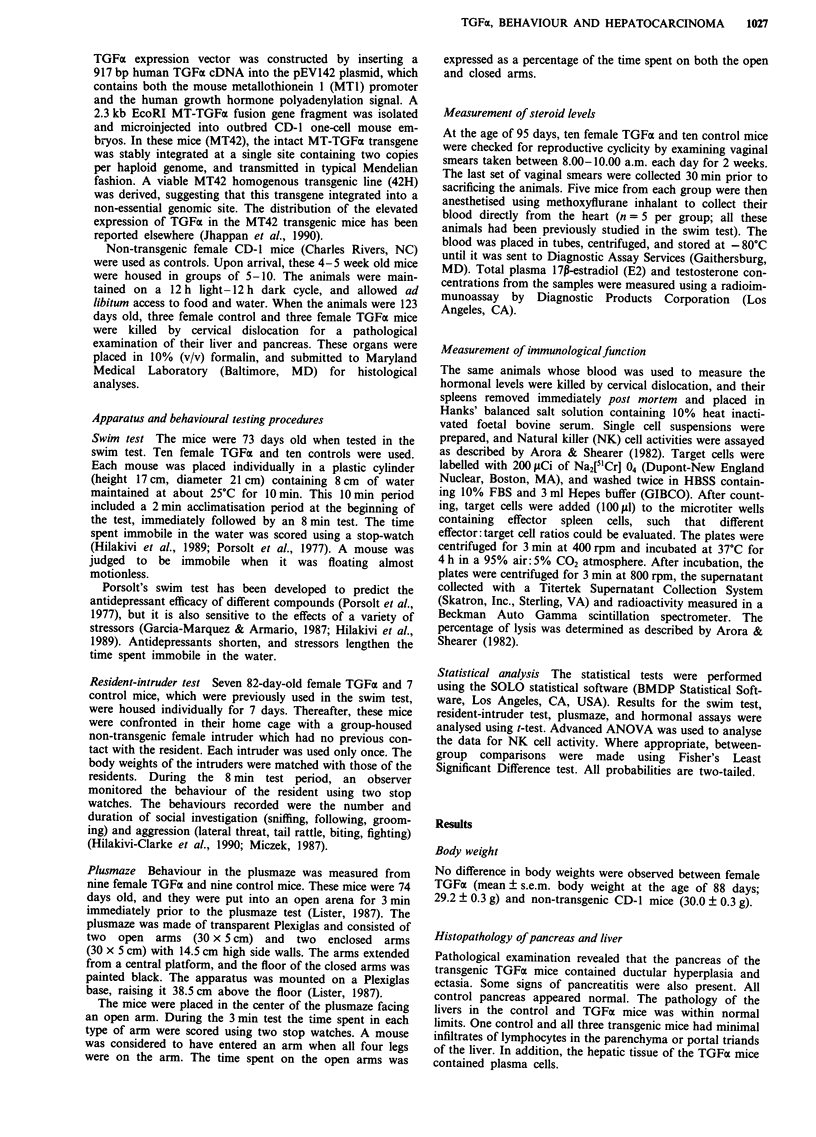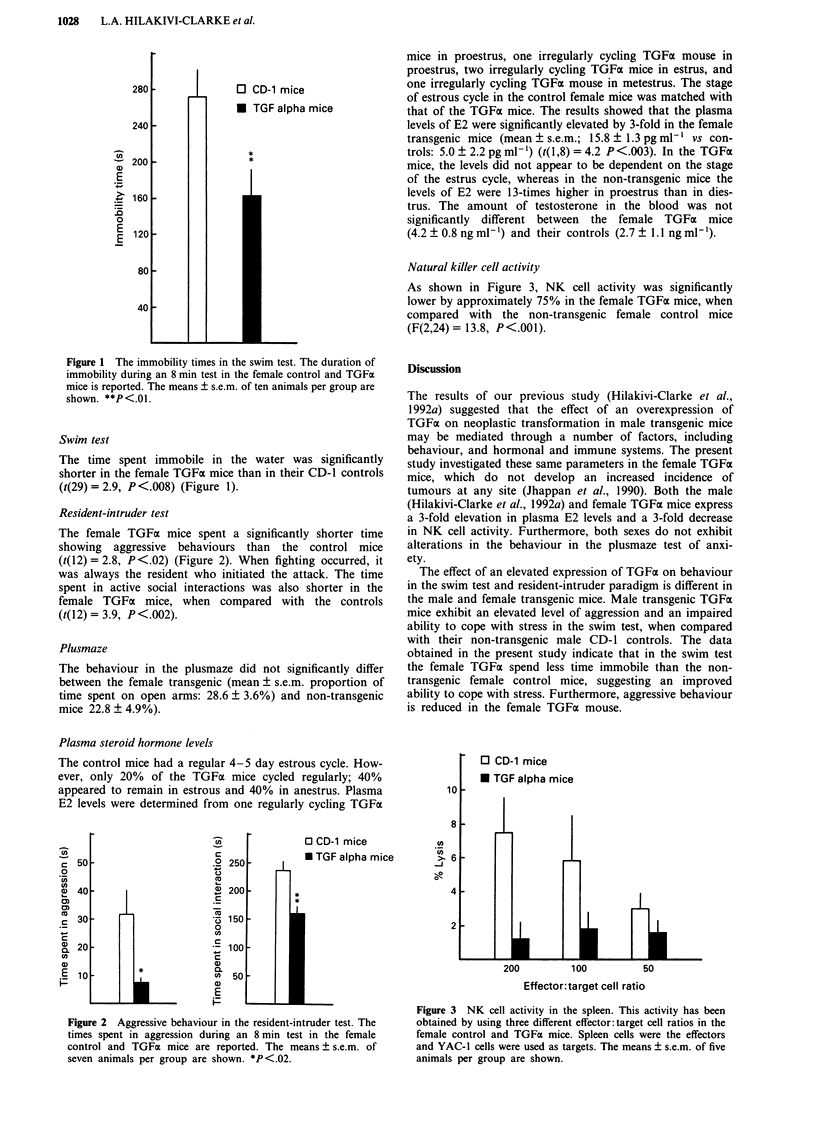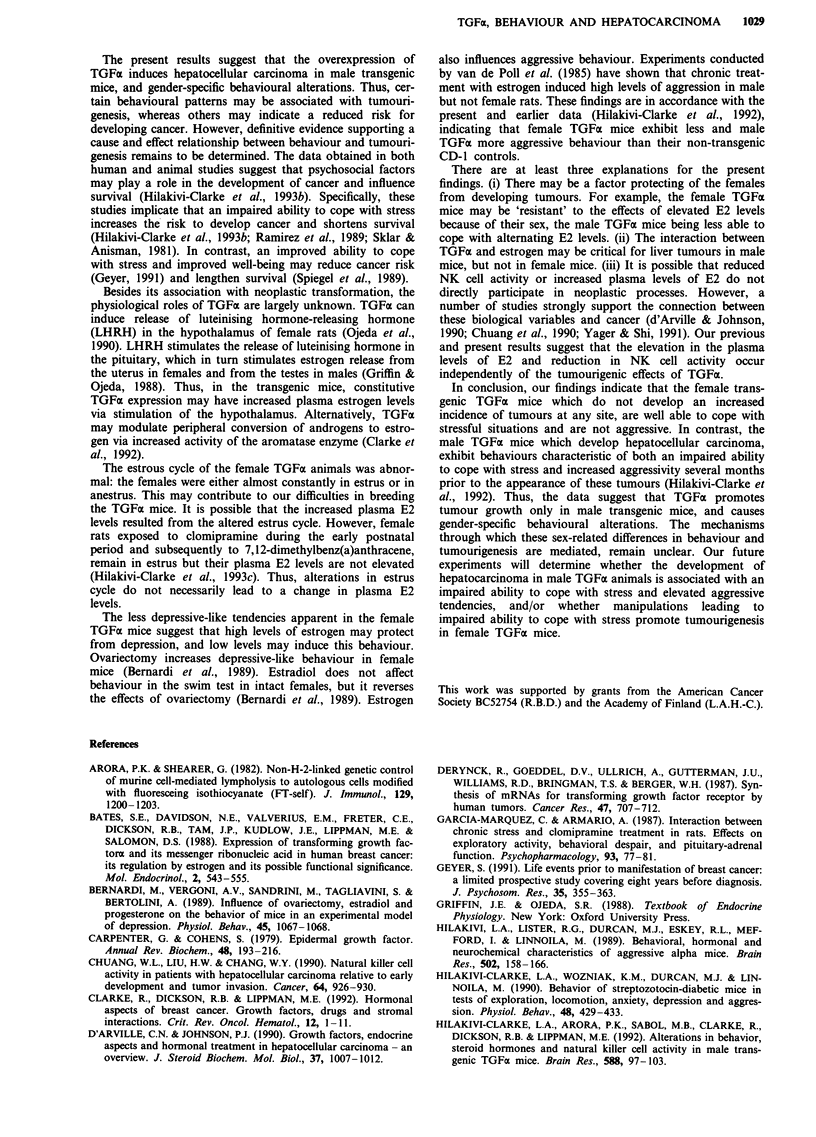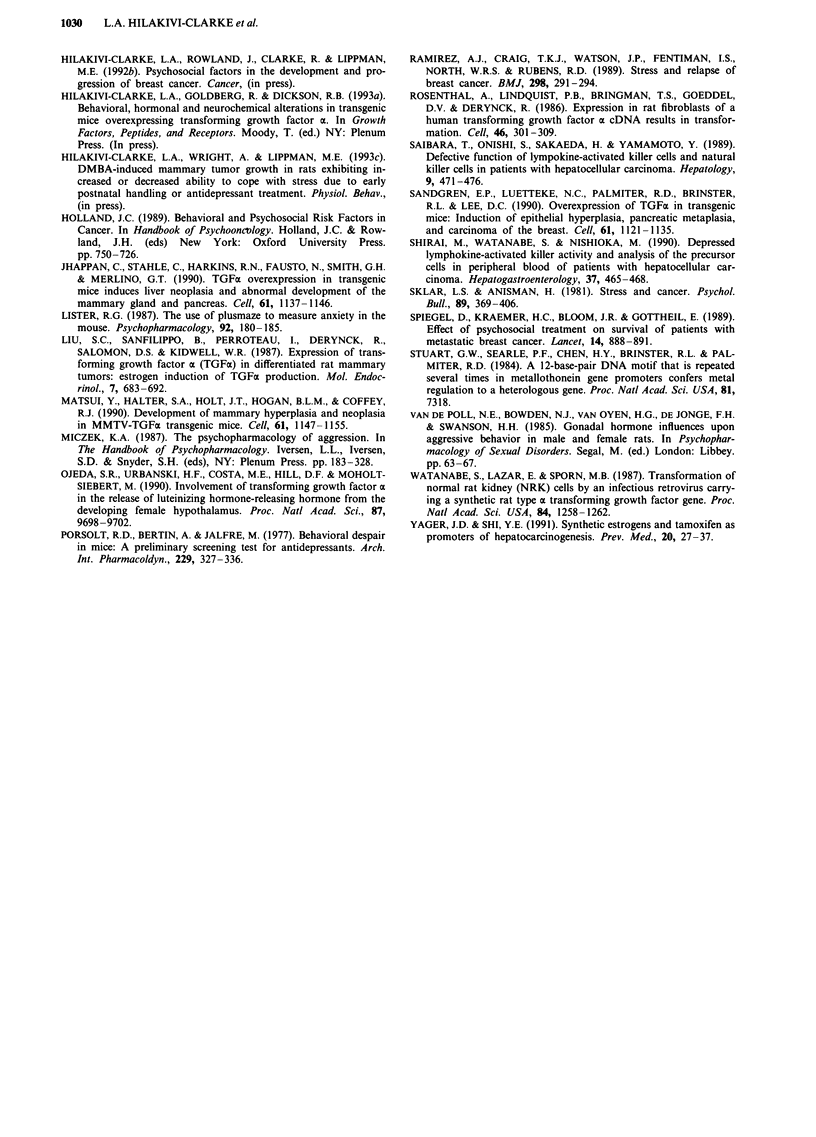# Opposing behavioural alterations in male and female transgenic TGF alpha mice: association with tumour susceptibility.

**DOI:** 10.1038/bjc.1993.188

**Published:** 1993-05

**Authors:** L. A. Hilakivi-Clarke, P. K. Arora, R. Clarke, A. Wright, M. E. Lippman, R. B. Dickson

**Affiliations:** Vincent T. Lombardi Cancer Research Center, Washington, DC 20007.

## Abstract

Psychosocial factors are thought to influence risk and survival from cancer. We have previously studied specific behaviours in transgenic male CD-1 MT42 mice, which overexpress the gene encoding human transforming growth factor alpha (TGF alpha) in multiple tissues, and which develop a high incidence of spontaneous hepatocellular carcinoma. The male TGF alpha mice spent a lengthened time immobile in the swim test, were highly aggressive, had increased plasma levels of 17 beta-estradiol (E2), and reduced natural killer (NK) cell activity. The female transgenic MT42 TGF alpha mice do not develop an increased rate of tumours at any site. We hypothesised that if the alterations in male TGF alpha mice are associated with their development of hepatocellular carcinomas, female TGF alpha should not show these alterations. The data in the present study indicate that female TGF alpha mice display shortened immobility in the swim test, suggesting an improved ability to cope with stress, and appear less aggressive in the resident-intruder test than non-transgenic female CD-1 mice. The female TGF alpha mice also exhibit a 3-fold increase in the plasma levels of E2, and a 3-fold increase in NK cell activity. These findings suggest that the elevated expression of TGF alpha in the transgenic mice is associated with gender-specific behavioural alterations, and the development of spontaneous hepatocellular tumours in the males. Furthermore, TGF alpha alters hormonal and immune parameters similarly in both sexes. It remains to be determined whether the development of hepatocarcinoma in the male TGF alpha animals is associated with an impaired ability to cope with stress and elevated aggressive tendencies and/or whether manipulations leading to an impaired ability to cope with stress will promote tumourigenesis in female TGF alpha mice.


*Br. J. Cancer* (1993), **67**, 1026–1030 © Macmillan Press Ltd., 1993

### Opposing behavioural alterations in male and female transgenic TGFα mice: association with tumour susceptibility

L.A. Hilakivi-Clarke[1,2], P.K. Arora[3], R. Clarke[1], A. Wright[1], M.E. Lippman[1] & R.B. Dickson[1]

[1]*Vincent T. Lombardi Cancer Research Center, and* [2]*Department of Psychiatry, Georgetown University Medical School, 3800 Reservoir Rd, N.W., Washington, DC 20007; and* [3]*Laboratory of Neuroscience NIDDK, 9000 Rockville Pike, Bethesda, Maryland 20892, USA.*

**Summary** Psychosocial factors are thought to influence risk and survival from cancer. We have previously studied specific behaviours in transgenic male CD-1 MT42 mice, which overexpress the gene encoding human transforming growth factor α (TGFα) in multiple tissues, and which develop a high incidence of spontaneous hepatocellular carcinoma. The male TGFα mice spent a lengthened time immobile in the swim test, were highly aggressive, had increased plasma levels of 17β-estradiol (E2), and reduced natural killer (NK) cell activity. The female transgenic MT42 TGFα mice do not develop an increased rate of tumours at any site. We hypothesised that if the alterations in male TGFα mice are associated with their development of hepatocellular carcinomas, female TGFα should not show these alterations. The data in the present study indicate that female TGFα mice display shortened immobility in the swim test, suggesting an improved ability to cope with stress, and appear less aggressive in the resident-intruder test than non-transgenic female CD-1 mice. The female TGFα mice also exhibit a 3-fold increase in the plasma levels of E2, and a 3-fold increase in NK cell activity.

These findings suggest that the elevated expression of TGFα in the transgenic mice is associated with gender-specific behavioural alterations, and the development of spontaneous hepatocellular tumours in the males. Furthermore, TGFα alters hormonal and immune parameters similarly in both sexes. It remains to be determined whether the development of hepatocarcinoma in the male TGFα animals is associated with an impaired ability to cope with stress and elevated aggressive tendencies and/or whether manipulations leading to an impaired ability to cope with stress will promote tumourigenesis in female TGFα mice.

Transforming growth factor α (TGFα) is a polypeptide which exhibits approximately 35% sequence homology to epidermal growth factor (EGF), and interacts with cells through the EGF receptor. Both TGFα and EGF can act as potent mitogens in a number of epithelial cell systems (Carpenter & Cohen, 1979). It has been postulated that TGFα plays an important role in neoplastic transformation, since it is highly expressed along with its receptor in many TGFα growth responsive tumour cells (Bates *et al*., 1988; Derynck *et al*., 1987; Rosenthal *et al*., 1986; Watanabe *et al*., 1987). The results of studies with transgenic CD-1 mice, in which chronic overexpression of TGFα is directed by the metallothionein (MT) promotor to multiple tissues throughout their life (Jhappan *et al*., 1990; Sandgren *et al*., 1990), have shown that male mice bearing a TGFα transgene develop a high incidence of spontaneous hepatocarcinomas at the age of 10–15 months (Jhappan *et al*., 1990). Other abnormalities in these animals (stomach, pancreas, etc.) have emerged as studies have proceeded.

Human and animal data suggest that an ability to cope with stress influences vulnerability to develop cancer (Hilakivi-Clarke *et al*., 1993*b*; Holland, 1989; Sklar & Anisman, 1981). Perturbations in natural killer (NK) cell activity (Chuang *et al*., 1990; Saibara *et al*., 1989; Shirai *et al*., 1990) and steroid hormone levels (d'Arville & Johnson, 1990; Yager & Shi, 1991) may mediate the effects of psychosocial factors in cancer. We have utilised transgenic male MT42 TGFα mice in studies assessing those behaviours associated with tumourigenesis (Hilakivi-Clarke *et al*., 1992*a*). When compared with age matched non-tran sgenic control CD-1 mice, 2–3-month-old male transgenic TGFα mice spent significantly longer times immobile in Porsolt's swim test, and in aggressive behaviour (Hilakivi-Clarke *et al*., 1992*a*). The male TGFα mice also exhibited a 25% lower NK cell activity, and a 4-fold increase in the plasma levels of 17β-estradiol (E2) than the controls (Hilakivi-Clarke *et al*., 1992). The results suggest that the effects of TGFα on hepatocellular carcinoma may be influenced by behaviour, and the immune and hormonal systems.

The present study investigated behavioural and biological parameters in the female transgenic MT42 TGFα mice. These female transgenic TGFα mice show an abnormal development of the mammary gland, but do not develop spontaneous tumours at an increased rate at any site (Jhappan *et al*., 1990). This is surprising, since TGFα appears to play a significant role in mammary tumourigenesis (Bates *et al*., 1988; Liu *et al*., 1987; Sandgren *et al*., 1990), and female mice in other transgenic TGFα models exhibit an increased incidence of mammary tumours (Matsui *et al*., 1990; Sandgren *et al*., 1990; Stuart, 1984). The expression of TGFα mRNA appears equally elevated in both sexes of MT42 mice (Hilakivi-Clarke *et al*., 1993*a*; Jhappan *et al*., 1990). To investigate behavioural changes associated with the overexpression of TGFα in the female mice, we utilised (i) Porsolt's swim test, which is thought to measure both depressive behaviour and an animal's ability to cope with stress (Garcia-Marquez & Armario, 1987; Hilakivi *et al*., 1989; Porsolt *et al*., 1977), (ii) the plusmaze test of anxiety (Lister, 1987), and (iii) the resident-intruder paradigm of aggression (Miczek, 1987). The NK cell activity, and plasma E2 and testosterone levels were also measured.

The results indicate that female transgenic TGFα mice show significantly shorter immobility in the swim test, and appear less aggressive than their non-transgenic CD-1 female controls. In marked contrast, the male TGFα mice develop hepatocellular tumours, exhibit a lengthened immobility in the swim test and are highly aggressive (Hilakivi-Clarke *et al*., 1992*a*). Thus, in addition to the differences in the tumourigenesis, overexpression of TGFα induces gender-specific behavioural alterations.

### Methods

### *Animals*

Mice of the CD-1 background were made transgenic for the growth factor TGFα (Jhappan *et al*., 1990) and provided by Dr Glenn Merlino (NCI, Frederick, MD). An inducible

Correspondence: L.A. Hilakivi-Clarke, Lombardi Cancer Research Center, Room S128, Georgetown University Medical School, 3800 Reservoir Rd, N.W., Washington, DC 20007, USA. Received 1 September 1992; and in revised form 14 January 1993.

TGFα expression vector was constructed by inserting a 917 bp human TGFα cDNA into the pEV142 plasmid, which contains both the mouse metallothionein 1 (MT1) promoter and the human growth hormone polyadenylation signal. A 2.3 kb EcoRI MT-TGFα fusion gene fragment was isolated and microinjected into outbred CD-1 one-cell mouse embryos. In these mice (MT42), the intact MT-TGFα transgene was stably integrated at a single site containing two copies per haploid genome, and transmitted in typical Mendelian fashion. A viable MT42 homogenous transgenic line (42H) was derived, suggesting that this transgene integrated into a non-essential genomic site. The distribution of the elevated expression of TGFα in the MT42 transgenic mice has been reported elsewhere (Jhappan *et al.*, 1990).

Non-transgenic female CD-1 mice (Charles Rivers, NC) were used as controls. Upon arrival, these 4–5 week old mice were housed in groups of 5–10. The animals were maintained on a 12 h light–12 h dark cycle, and allowed *ad libitum* access to food and water. When the animals were 123 days old, three female control and three female TGFα mice were killed by cervical dislocation for a pathological examination of their liver and pancreas. These organs were placed in 10% (v/v) formalin, and submitted to Maryland Medical Laboratory (Baltimore, MD) for histological analyses.

### *Apparatus and behavioural testing procedures*

*Swim test* The mice were 73 days old when tested in the swim test. Ten female TGFα and ten controls were used. Each mouse was placed individually in a plastic cylinder (height 17 cm, diameter 21 cm) containing 8 cm of water maintained at about 25°C for 10 min. This 10 min period included a 2 min acclimatisation period at the beginning of the test, immediately followed by an 8 min test. The time spent immobile in the water was scored using a stop-watch (Hilakivi *et al.*, 1989; Porsolt *et al.*, 1977). A mouse was judged to be immobile when it was floating almost motionless.

Porsolt's swim test has been developed to predict the antidepressant efficacy of different compounds (Porsolt *et al.*, 1977), but it is also sensitive to the effects of a variety of stressors (Garcia-Marquez & Armario, 1987; Hilakivi *et al.*, 1989). Antidepressants shorten, and stressors lengthen the time spent immobile in the water.

*Resident-intruder test* Seven 82-day-old female TGFα and 7 control mice, which were previously used in the swim test, were housed individually for 7 days. Thereafter, these mice were confronted in their home cage with a group-housed non-transgenic female intruder which had no previous contact with the resident. Each intruder was used only once. The body weights of the intruders were matched with those of the residents. During the 8 min test period, an observer monitored the behaviour of the resident using two stop watches. The behaviours recorded were the number and duration of social investigation (sniffing, following, grooming) and aggression (lateral threat, tail rattle, biting, fighting) (Hilakivi-Clarke *et al.*, 1990; Miczek, 1987).

*Plusmaze* Behaviour in the plusmaze was measured from nine female TGFα and nine control mice. These mice were 74 days old, and they were put into an open arena for 3 min immediately prior to the plusmaze test (Lister, 1987). The plusmaze was made of transparent Plexiglas and consisted of two open arms (30 × 5 cm) and two enclosed arms (30 × 5 cm) with 14.5 cm high side walls. The arms extended from a central platform, and the floor of the closed arms was painted black. The apparatus was mounted on a Plexiglas base, raising it 38.5 cm above the floor (Lister, 1987).

The mice were placed in the center of the plusmaze facing an open arm. During the 3 min test the time spent in each type of arm were scored using two stop watches. A mouse was considered to have entered an arm when all four legs were on the arm. The time spent on the open arms was expressed as a percentage of the time spent on both the open and closed arms.

### *Measurement of steroid levels*

At the age of 95 days, ten female TGFα and ten control mice were checked for reproductive cyclicity by examining vaginal smears taken between 8.00–10.00 a.m. each day for 2 weeks. The last set of vaginal smears were collected 30 min prior to sacrificing the animals. Five mice from each group were then anesthetised using methoxyflurane inhalant to collect their blood directly from the heart ($n = 5$ per group; all these animals had been previously studied in the swim test). The blood was placed in tubes, centrifuged, and stored at −80°C until it was sent to Diagnostic Assay Services (Gaithersburg, MD). Total plasma 17β-estradiol (E2) and testosterone concentrations from the samples were measured using a radioimmunoassay by Diagnostic Products Corporation (Los Angeles, CA).

### *Measurement of immunological function*

The same animals whose blood was used to measure the hormonal levels were killed by cervical dislocation, and their spleens removed immediately *post mortem* and placed in Hanks' balanced salt solution containing 10% heat inactivated foetal bovine serum. Single cell suspensions were prepared, and Natural killer (NK) cell activities were assayed as described by Arora & Shearer (1982). Target cells were labelled with 200 μCi of $Na_2[^{51}Cr]\ 0_4$ (Dupont-New England Nuclear, Boston, MA), and washed twice in HBSS containing 10% FBS and 3 ml Hepes buffer (GIBCO). After counting, target cells were added (100 μl) to the microtiter wells containing effector spleen cells, such that different effector: target cell ratios could be evaluated. The plates were centrifuged for 3 min at 400 rpm and incubated at 37°C for 4 h in a 95% air: 5% $CO_2$ atmosphere. After incubation, the plates were centrifuged for 3 min at 800 rpm, the supernatant collected with a Titertek Supernatant Collection System (Skatron, Inc., Sterling, VA) and radioactivity measured in a Beckman Auto Gamma scintillation spectrometer. The percentage of lysis was determined as described by Arora & Shearer (1982).

*Statistical analysis* The statistical tests were performed using the SOLO statistical software (BMDP Statistical Software, Los Angeles, CA, USA). Results for the swim test, resident-intruder test, plusmaze, and hormonal assays were analysed using *t*-test. Advanced ANOVA was used to analyse the data for NK cell activity. Where appropriate, between-group comparisons were made using Fisher's Least Significant Difference test. All probabilities are two-tailed.

### Results

### *Body weight*

No difference in body weights were observed between female TGFα (mean ± s.e.m. body weight at the age of 88 days; 29.2 ± 0.3 g) and non-transgenic CD-1 mice (30.0 ± 0.3 g).

### *Histopathology of pancreas and liver*

Pathological examination revealed that the pancreas of the transgenic TGFα mice contained ductular hyperplasia and ectasia. Some signs of pancreatitis were also present. All control pancreas appeared normal. The pathology of the livers in the control and TGFα mice was within normal limits. One control and all three transgenic mice had minimal infiltrates of lymphocytes in the parenchyma or portal triands of the liver. In addition, the hepatic tissue of the TGFα mice contained plasma cells.

**Figure 1** The immobility times in the swim test. The duration of immobility during an 8 min test in the female control and TGFα mice is reported. The means ± s.e.m. of ten animals per group are shown. $^{**}P<.01$.

### *Swim test*

The time spent immobile in the water was significantly shorter in the female TGFα mice than in their CD-1 controls ($t(29) = 2.9$, $P<.008$) (Figure 1).

### *Resident-intruder test*

The female TGFα mice spent a significantly shorter time showing aggressive behaviours than the control mice ($t(12) = 2.8$, $P<.02$) (Figure 2). When fighting occurred, it was always the resident who initiated the attack. The time spent in active social interactions was also shorter in the female TGFα mice, when compared with the controls ($t(12) = 3.9$, $P<.002$).

### *Plusmaze*

The behaviour in the plusmaze did not significantly differ between the female transgenic (mean ± s.e.m. proportion of time spent on open arms: 28.6 ± 3.6%) and non-transgenic mice 22.8 ± 4.9%).

### *Plasma steroid hormone levels*

The control mice had a regular 4–5 day estrous cycle. However, only 20% of the TGFα mice cycled regularly; 40% appeared to remain in estrous and 40% in anestrus. Plasma E2 levels were determined from one regularly cycling TGFα mice in proestrus, one irregularly cycling TGFα mouse in proestrus, two irregularly cycling TGFα mice in estrus, and one irregularly cycling TGFα mouse in metestrus. The stage of estrous cycle in the control female mice was matched with that of the TGFα mice. The results showed that the plasma levels of E2 were significantly elevated by 3-fold in the female transgenic mice (mean ± s.e.m.; $15.8 \pm 1.3\ \mathrm{pg\ ml^{-1}}$ *vs* controls: $5.0 \pm 2.2\ \mathrm{pg\ ml^{-1}}$) ($t(1,8) = 4.2$ $P<.003$). In the TGFα mice, the levels did not appear to be dependent on the stage of the estrus cycle, whereas in the non-transgenic mice the levels of E2 were 13-times higher in proestrus than in diestrus. The amount of testosterone in the blood was not significantly different between the female TGFα mice ($4.2 \pm 0.8\ \mathrm{ng\ ml^{-1}}$) and their controls ($2.7 \pm 1.1\ \mathrm{ng\ ml^{-1}}$).

**Figure 2** Aggressive behaviour in the resident-intruder test. The times spent in aggression during an 8 min test in the female control and TGFα mice are reported. The means ± s.e.m. of seven animals per group are shown. $^{*}P<.02$.

### *Natural killer cell activity*

As shown in Figure 3, NK cell activity was significantly lower by approximately 75% in the female TGFα mice, when compared with the non-transgenic female control mice ($F(2,24) = 13.8$, $P<.001$).

### Discussion

The results of our previous study (Hilakivi-Clarke *et al.*, 1992*a*) suggested that the effect of an overexpression of TGFα on neoplastic transformation in male transgenic mice may be mediated through a number of factors, including behaviour, and hormonal and immune systems. The present study investigated these same parameters in the female TGFα mice, which do not develop an increased incidence of tumours at any site (Jhappan *et al.*, 1990). Both the male (Hilakivi-Clarke *et al.*, 1992*a*) and female TGFα mice express a 3-fold elevation in plasma E2 levels and a 3-fold decrease in NK cell activity. Furthermore, both sexes do not exhibit alterations in the behaviour in the plusmaze test of anxiety.

The effect of an elevated expression of TGFα on behaviour in the swim test and resident-intruder paradigm is different in the male and female transgenic mice. Male transgenic TGFα mice exhibit an elevated level of aggression and an impaired ability to cope with stress in the swim test, when compared with their non-transgenic male CD-1 controls. The data obtained in the present study indicate that in the swim test the female TGFα spend less time immobile than the non-transgenic female control mice, suggesting an improved ability to cope with stress. Furthermore, aggressive behaviour is reduced in the female TGFα mouse.

**Figure 3** NK cell activity in the spleen. This activity has been obtained by using three different effector:target cell ratios in the female control and TGFα mice. Spleen cells were the effectors and YAC-1 cells were used as targets. The means ± s.e.m. of five animals per group are shown.

The present results suggest that the overexpression of TGFα induces hepatocellular carcinoma in male transgenic mice, and gender-specific behavioural alterations. Thus, certain behavioural patterns may be associated with tumourigenesis, whereas others may indicate a reduced risk for developing cancer. However, definitive evidence supporting a cause and effect relationship between behaviour and tumourigenesis remains to be determined. The data obtained in both human and animal studies suggest that psychosocial factors may play a role in the development of cancer and influence survival (Hilakivi-Clarke *et al.*, 1993*b*). Specifically, these studies implicate that an impaired ability to cope with stress increases the risk to develop cancer and shortens survival (Hilakivi-Clarke *et al.*, 1993*b*; Ramirez *et al.*, 1989; Sklar & Anisman, 1981). In contrast, an improved ability to cope with stress and improved well-being may reduce cancer risk (Geyer, 1991) and lengthen survival (Spiegel *et al.*, 1989).

Besides its association with neoplastic transformation, the physiological roles of TGFα are largely unknown. TGFα can induce release of luteinising hormone-releasing hormone (LHRH) in the hypothalamus of female rats (Ojeda *et al.*, 1990). LHRH stimulates the release of luteinising hormone in the pituitary, which in turn stimulates estrogen release from the uterus in females and from the testes in males (Griffin & Ojeda, 1988). Thus, in the transgenic mice, constitutive TGFα expression may have increased plasma estrogen levels via stimulation of the hypothalamus. Alternatively, TGFα may modulate peripheral conversion of androgens to estrogen via increased activity of the aromatase enzyme (Clarke *et al.*, 1992).

The estrous cycle of the female TGFα animals was abnormal: the females were either almost constantly in estrus or in anestrus. This may contribute to our difficulties in breeding the TGFα mice. It is possible that the increased plasma E2 levels resulted from the altered estrus cycle. However, female rats exposed to clomipramine during the early postnatal period and subsequently to 7,12-dimethylbenz(a)anthracene, remain in estrus but their plasma E2 levels are not elevated (Hilakivi-Clarke *et al.*, 1993*c*). Thus, alterations in estrus cycle do not necessarily lead to a change in plasma E2 levels.

The less depressive-like tendencies apparent in the female TGFα mice suggest that high levels of estrogen may protect from depression, and low levels may induce this behaviour. Ovariectomy increases depressive-like behaviour in female mice (Bernardi *et al.*, 1989). Estradiol does not affect behaviour in the swim test in intact females, but it reverses the effects of ovariectomy (Bernardi *et al.*, 1989). Estrogen also influences aggressive behaviour. Experiments conducted by van de Poll *et al.* (1985) have shown that chronic treatment with estrogen induced high levels of aggression in male but not female rats. These findings are in accordance with the present and earlier data (Hilakivi-Clarke *et al.*, 1992), indicating that female TGFα mice exhibit less and male TGFα more aggressive behaviour than their non-transgenic CD-1 controls.

There are at least three explanations for the present findings. (i) There may be a factor protecting of the females from developing tumours. For example, the female TGFα mice may be 'resistant' to the effects of elevated E2 levels because of their sex, the male TGFα mice being less able to cope with alternating E2 levels. (ii) The interaction between TGFα and estrogen may be critical for liver tumours in male mice, but not in female mice. (iii) It is possible that reduced NK cell activity or increased plasma levels of E2 do not directly participate in neoplastic processes. However, a number of studies strongly support the connection between these biological variables and cancer (d'Arville & Johnson, 1990; Chuang *et al.*, 1990; Yager & Shi, 1991). Our previous and present results suggest that the elevation in the plasma levels of E2 and reduction in NK cell activity occur independently of the tumourigenic effects of TGFα.

In conclusion, our findings indicate that the female transgenic TGFα mice which do not develop an increased incidence of tumours at any site, are well able to cope with stressful situations and are not aggressive. In contrast, the male TGFα mice which develop hepatocellular carcinoma, exhibit behaviours characteristic of both an impaired ability to cope with stress and increased aggressivity several months prior to the appearance of these tumours (Hilakivi-Clarke *et al.*, 1992). Thus, the data suggest that TGFα promotes tumour growth only in male transgenic mice, and causes gender-specific behavioural alterations. The mechanisms through which these sex-related differences in behaviour and tumourigenesis are mediated, remain unclear. Our future experiments will determine whether the development of hepatocarcinoma in male TGFα animals is associated with an impaired ability to cope with stress and elevated aggressive tendencies, and/or whether manipulations leading to impaired ability to cope with stress promote tumourigenesis in female TGFα mice.

This work was supported by grants from the American Cancer Society BC52754 (R.B.D.) and the Academy of Finland (L.A.H.-C.).

### References

ARORA, P.K. & SHEARER, G. (1982). Non-H-2-linked genetic control of murine cell-mediated lympholysis to autologous cells modified with fluoresceing isothiocyanate (FT-self). *J. Immunol.*, **129**, 1200–1203.

BATES, S.E., DAVIDSON, N.E., VALVERIUS, E.M., FRETER, C.E., DICKSON, R.B., TAM, J.P., KUDLOW, J.E., LIPPMAN, M.E. & SALOMON, D.S. (1988). Expression of transforming growth factorα and its messenger ribonucleic acid in human breast cancer: its regulation by estrogen and its possible functional significance. *Mol. Endocrinol.*, **2**, 543–555.

BERNARDI, M., VERGONI, A.V., SANDRINI, M., TAGLIAVINI, S. & BERTOLINI, A. (1989). Influence of ovariectomy, estradiol and progesterone on the behavior of mice in an experimental model of depression. *Physiol. Behav.*, **45**, 1067–1068.

CARPENTER, G. & COHENS, S. (1979). Epidermal growth factor. *Annual Rev. Biochem.*, **48**, 193–216.

CHUANG, W.L., LIU, H.W. & CHANG, W.Y. (1990). Natural killer cell activity in patients with hepatocellular carcinoma relative to early development and tumor invasion. *Cancer*, **64**, 926–930.

CLARKE, R., DICKSON, R.B. & LIPPMAN, M.E. (1992). Hormonal aspects of breast cancer. Growth factors, drugs and stromal interactions. *Crit. Rev. Oncol. Hematol.*, **12**, 1–11.

D'ARVILLE, C.N. & JOHNSON, P.J. (1990). Growth factors, endocrine aspects and hormonal treatment in hepatocellular carcinoma – an overview. *J. Steroid Biochem. Mol. Biol.*, **37**, 1007–1012.

DERYNCK, R., GOEDDEL, D.V., ULLRICH, A., GUTTERMAN, J.U., WILLIAMS, R.D., BRINGMAN, T.S. & BERGER, W.H. (1987). Synthesis of mRNAs for transforming growth factor receptor by human tumors. *Cancer Res.*, **47**, 707–712.

GARCIA-MARQUEZ, C. & ARMARIO, A. (1987). Interaction between chronic stress and clomipramine treatment in rats. Effects on exploratory activity, behavioral despair, and pituitary-adrenal function. *Psychopharmacology*, **93**, 77–81.

GEYER, S. (1991). Life events prior to manifestation of breast cancer: a limited prospective study covering eight years before diagnosis. *J. Psychosom. Res.*, **35**, 355–363.

GRIFFIN, J.E. & OJEDA, S.R. (1988). *Textbook of Endocrine Physiology*. New York: Oxford University Press.

HILAKIVI, L.A., LISTER, R.G., DURCAN, M.J., ESKEY, R.L., MEFFORD, I. & LINNOILA, M. (1989). Behavioral, hormonal and neurochemical characteristics of aggressive alpha mice. *Brain Res.*, **502**, 158–166.

HILAKIVI-CLARKE, L.A., WOZNIAK, K.M., DURCAN, M.J. & LINNOILA, M. (1990). Behavior of streptozotocin-diabetic mice in tests of exploration, locomotion, anxiety, depression and aggression. *Physiol. Behav.*, **48**, 429–433.

HILAKIVI-CLARKE, L.A., ARORA, P.K., SABOL, M.B., CLARKE, R., DICKSON, R.B. & LIPPMAN, M.E. (1992). Alterations in behavior, steroid hormones and natural killer cell activity in male transgenic TGFα mice. *Brain Res.*, **588**, 97–103.

HILAKIVI-CLARKE, L.A., ROWLAND, J., CLARKE, R. & LIPPMAN, M.E. (1992*b*). Psychosocial factors in the development and progression of breast cancer. *Cancer*, (in press).

HILAKIVI-CLARKE, L.A., GOLDBERG, R. & DICKSON, R.B. (1993*a*). Behavioral, hormonal and neurochemical alterations in transgenic mice overexpressing transforming growth factor α. In *Growth Factors, Peptides, and Receptors*. Moody, T. (ed.) NY: Plenum Press. (In press).

HILAKIVI-CLARKE, L.A., WRIGHT, A. & LIPPMAN, M.E. (1993*c*). DMBA-induced mammary tumor growth in rats exhibiting increased or decreased ability to cope with stress due to early postnatal handling or antidepressant treatment. *Physiol. Behav.*, (in press).

HOLLAND, J.C. (1989). Behavioral and Psychosocial Risk Factors in Cancer. In *Handbook of Psychooncology*. Holland, J.C. & Rowland, J.H. (eds) New York: Oxford University Press. pp. 750–726.

JHAPPAN, C., STAHLE, C., HARKINS, R.N., FAUSTO, N., SMITH, G.H. & MERLINO, G.T. (1990). TGFα overexpression in transgenic mice induces liver neoplasia and abnormal development of the mammary gland and pancreas. *Cell*, **61**, 1137–1146.

LISTER, R.G. (1987). The use of plusmaze to measure anxiety in the mouse. *Psychopharmacology*, **92**, 180–185.

LIU, S.C., SANFILIPPO, B., PERROTEAU, I., DERYNCK, R., SALOMON, D.S. & KIDWELL, W.R. (1987). Expression of transforming growth factor α (TGFα) in differentiated rat mammary tumors: estrogen induction of TGFα production. *Mol. Endocrinol.*, **7**, 683–692.

MATSUI, Y., HALTER, S.A., HOLT, J.T., HOGAN, B.L.M., & COFFEY, R.J. (1990). Development of mammary hyperplasia and neoplasia in MMTV-TGFα transgenic mice. *Cell*, **61**, 1147–1155.

MICZEK, K.A. (1987). The psychopharmacology of aggression. In *The Handbook of Psychopharmacology*. Iversen, L.L., Iversen, S.D. & Snyder, S.H. (eds), NY: Plenum Press. pp. 183–328.

OJEDA, S.R., URBANSKI, H.F., COSTA, M.E., HILL, D.F. & MOHOLT-SIEBERT, M. (1990). Involvement of transforming growth factor α in the release of luteinizing hormone-releasing hormone from the developing female hypothalamus. *Proc. Natl Acad. Sci.*, **87**, 9698–9702.

PORSOLT, R.D., BERTIN, A. & JALFRE, M. (1977). Behavioral despair in mice: A preliminary screening test for antidepressants. *Arch. Int. Pharmacoldyn.*, **229**, 327–336.

RAMIREZ, A.J., CRAIG, T.K.J., WATSON, J.P., FENTIMAN, I.S., NORTH, W.R.S. & RUBENS, R.D. (1989). Stress and relapse of breast cancer. *BMJ*, **298**, 291–294.

ROSENTHAL, A., LINDQUIST, P.B., BRINGMAN, T.S., GOEDDEL, D.V. & DERYNCK, R. (1986). Expression in rat fibroblasts of a human transforming growth factor α cDNA results in transformation. *Cell*, **46**, 301–309.

SAIBARA, T., ONISHI, S., SAKAEDA, H. & YAMAMOTO, Y. (1989). Defective function of lympokine-activated killer cells and natural killer cells in patients with hepatocellular carcinoma. *Hepatology*, **9**, 471–476.

SANDGREN, E.P., LUETTEKE, N.C., PALMITER, R.D., BRINSTER, R.L. & LEE, D.C. (1990). Overexpression of TGFα in transgenic mice: Induction of epithelial hyperplasia, pancreatic metaplasia, and carcinoma of the breast. *Cell*, **61**, 1121–1135.

SHIRAI, M., WATANABE, S. & NISHIOKA, M. (1990). Depressed lymphokine-activated killer activity and analysis of the precursor cells in peripheral blood of patients with hepatocellular carcinoma. *Hepatogastroenterology*, **37**, 465–468.

SKLAR, L.S. & ANISMAN, H. (1981). Stress and cancer. *Psychol. Bull.*, **89**, 369–406.

SPIEGEL, D., KRAEMER, H.C., BLOOM, J.R. & GOTTHEIL, E. (1989). Effect of psychosocial treatment on survival of patients with metastatic breast cancer. *Lancet*, **14**, 888–891.

STUART, G.W., SEARLE, P.F., CHEN, H.Y., BRINSTER, R.L. & PALMITER, R.D. (1984). A 12-base-pair DNA motif that is repeated several times in metallothonein gene promoters confers metal regulation to a heterologous gene. *Proc. Natl Acad. Sci. USA*, **81**, 7318.

VAN DE POLL, N.E., BOWDEN, N.J., VAN OYEN, H.G., DE JONGE, F.H. & SWANSON, H.H. (1985). Gonadal hormone influences upon aggressive behavior in male and female rats. In *Psychopharmacology of Sexual Disorders*. Segal, M. (ed.) London: Libbey. pp. 63–67.

WATANABE, S., LAZAR, E. & SPORN, M.B. (1987). Transformation of normal rat kidney (NRK) cells by an infectious retrovirus carrying a synthetic rat type α transforming growth factor gene. *Proc. Natl Acad. Sci. USA*, **84**, 1258–1262.

YAGER, J.D. & SHI, Y.E. (1991). Synthetic estrogens and tamoxifen as promoters of hepatocarcinogenesis. *Prev. Med.*, **20**, 27–37.